# Relationship between hand grip and pinch strength, body composition, pain threshold, and anxiety in dentists

**DOI:** 10.1007/s11845-025-03941-4

**Published:** 2025-03-28

**Authors:** Sena Gizem Arslan, Abdurrahim Yildiz, Birgul Dingirdan Gultekinler

**Affiliations:** 1 Physiotherapy and Rehabilitation Department, Faculty of Health Sciences, Sakarya University of Applied Sciences, Sakarya, Turkey; 2 Physiotherapy and Rehabilitation Application and Research Center, Sakarya University of Applied Sciences, Sakarya, Turkey

**Keywords:** Anxiety, Dentist, Grip strength, Pain, Pinch, Pinch strength

## Abstract

**Background:**

Work-related musculoskeletal disorders are commonly observed across various occupational groups.

**Aim:**

The aim of this study was to evaluate the relationships between hand grip and pinch strength, body composition, pain threshold, and anxiety levels in dentists.

**Methods:**

The study included 49 dentists working at Sakarya Oral and Dental Health Hospital. Participants’ grip strength, including pinch and gross grip, was assessed using the Jamar hand dynamometer, while the muscle strength of the forearm flexor and extensor muscles was measured with a handheld dynamometer. Anxiety levels were evaluated using the Beck Anxiety Inventory, and pain threshold was assessed using an algometer.

**Results:**

A positive and significant correlation was found between body mass index (BMI) and dominant and non-dominant hand grip strength (*r* = 0.485, *p* = 0.003; *r* = 0.501, *p* = 0.002). BMI also showed a strong relationship with dominant and non-dominant finger strength (*r* = 0.511, *p* < 0.001; *r* = 0.557, *p* < 0.001). A negative correlation was found between Beck Anxiety Score and physical strength parameters, especially non-dominant hand grip strength (*r* = 0.619, *p* = 0.005) and dominant hand grip strength (*r* =  − 0.512, *p* = 0.025) and anxiety levels. Significant positive correlations were found between pain threshold and physical strength parameters, especially non-dominant wrist extensor strength (*r* = 0.283, *p* = 0.049) and dominant hand grip strength (*r* = 0.408, *p* = 0.015) which were found to increase pain threshold.

**Conclusions:**

Physical and psychological factors have an impact on occupational performance, especially in occupations that require prolonged use of the hands and wrists, such as dentistry.

**Trial registration:**

This study is prospectively registered at NCT06721117 (http://clinicaltrials.gov).

## Introduction

Work-related musculoskeletal disorders can affect various occupational groups. Among these occupational groups are dentists. The postures adopted by dentists while performing their professional duties can contribute to the development of musculoskeletal disorders. As the duration spent in these positions increases, the severity of the problems may also escalate [1].

The functionality of the hand is highly significant in dentistry [2]. The primary indicators of hand function are sufficient pinch and gross grip strength. Various factors influence pinch and gross grip strength, including gender, anthropometric characteristics, occupational load, and age [3, 4]. Demographic characteristics such as weight and body mass index (BMI), as well as physical attributes such as hand and pinch length, palm width, wrist circumference, and forearm circumference, have been found to be associated with grip strength [2, 5]. Individuals with greater demographic characteristics such as weight and BMI, as well as physical attributes such as hand and pinch length, palm width, wrist circumference, and forearm circumference, are expected to have higher grip strength [2]. Dentists, by the nature of their profession, frequently engage in tasks requiring high precision and frequent use of the wrist and fine motor skills. Due to their occupational duties and working environments, the mechanical load on the pinch joints may vary [6]. Additionally, the functions most frequently performed by dentists may vary depending on their dominant hand. For example, dentists with right-hand dominance often use their thumb, index, and middle finger pinches intensively in the right hand, while the left hand primarily serves as supportive role and remains in a static position [6].

Dentists frequently perform repetitive activities involving the wrist and pinches. They also utilize various instruments during their work. The repetitive and frequent use of the upper extremity may lead to differences in muscle activation in the upper extremity and neck regions. Studies examining EMG activations in dentists’ working postures have shown that the extensor carpi radialis and infraspinatus muscles exhibit high activation levels in the dominant extremity, while the trapezius muscle demonstrates high activation levels on the non-dominant side [2]. In dentists, occupational activities often involve repetitive and prolonged wrist flexion/extension, forearm rotation, elbow flexion, thumb hyperflexion, neck flexion, shoulder elevation, and shoulder abduction. Additionally, repeated and sustained tight gripping of professional instruments is required. All these factors place additional strain on the musculoskeletal system of dentists, putting them at a disadvantage compared to other occupational groups. Musculoskeletal problems often arise during the precise physical activities performed by dentists in their profession, and as the duration of exposure increases, these issues may become persistent [7, 8].

Another reason dentists are exposed to improper asymmetrical postures is the small size of their working area (the oral cavity). Prolonged static work in non-ergonomic asymmetric positions can further exacerbate musculoskeletal problems [9]. Working conditions involving prolonged head lateral flexion and arms positioned away from the body increase the risk of occupational musculoskeletal disorders. Mechanical problems in the head, neck, and back regions are more frequently observed under such conditions [9].

The working conditions required by the dental profession impact dentists both physically and mentally [10]. The working conditions demanded by the profession comprehensively affect the physical and mental health of dentists, ultimately resulting in burnout, anxiety, and depression [10]. Anxiety and depression affect not only dentistry but also the working population [10, 11]. Considering the impact of mental health on the working population and healthcare professionals, addressing these issues and the associated risk factors is of significant importance [10]. Evaluating anxiety levels in dentists, a professional group where musculoskeletal problems are commonly observed, is also of significant importance.

This study offers a unique perspective by addressing the musculoskeletal problems commonly seen in dentists not only from a biomechanical point of view but also with psychosocial factors. Repetitive movements, static postures, and fine motor movements requiring manual dexterity may lead to changes in muscle strength and pain over time. In addition, it is thought that occupational stress as well as physical loading may have an effect on musculoskeletal health. This study aims to contribute to occupational health strategies by revealing how hand functions and muscle strength interact with ergonomic risk factors. In this direction, it is aimed to evaluate the relationships between hand grip and pinch strength, body composition, pain threshold, and anxiety levels in dentists.

## Materials and methods

### Study design

This is a cross-sectional, single-blind study with different people assessing the participants and interpreting the results. This trial was approved by the Ethics Committee at Sakarya University of Applied Science, Turkey (approval date: 13.06.2024, decision no: 45/ 48) and was conducted in accordance with the principles of the Declaration of Helsinki. Eligible participants received written information and provided informed consent before participation.

### Participants and selection

The study was conducted with dentists working at Sakarya Oral and Dental Health Hospital in November and December 2024. Dentists with 1 year or more professional experience were included in the study. Those who had received physical therapy from any area in the last 6 months or were currently receiving physical therapy, those with congenital musculoskeletal deformity, those with neurological or rheumatic diseases, and those who had surgery due to musculoskeletal disorders were excluded from the study. Sixty dentists were reached in the study, but 11 of them did not meet the inclusion criteria. In total, 49 dentists were evaluated and were included in the statistical analysis. The sample size was determined using the “G*power sample size calculator” [12]. The sample size was calculated as 42 subjects using “pinch grip strength’’ design, four repeated measures with a power of 0.95% (*α* = 0.05, *β* = 0.05), and an effect size of 1.05 [13].

### Outcome measurements

In the study, demographic information form was used to assess demographic information, Jamar Hand Dynamometer to assess hand grip strength, pinch metre device to assess pinch grip strength, Nicholas Manual Dynamometer to assess forearm muscle strength, algometer device to assess pain level, and Beck Anxiety Scale to determine anxiety level.

#### Demographic information form

It was prepared to record the sociodemographic characteristics of participants. With this form, age, gender, weight, height, BMI, smoking, alcohol use, and chronic disease information was obtained.

#### Grip strength assessment

The Jamar Hand Dynamometer, recommended by the American Hand Therapists Association and accepted as the gold standard in many studies due to its high validity and reliability, was used to measure hand grip strength. A pinch metre (Baseline Mechanical Pinch Gauge) was used to measure fine grip strength. The measurement of hand grip and pinch grip strength was made in the recommended standard position of sitting, shoulder adduction and neutral rotation, elbow 90 degrees flexed, forearm in mid-rotation and supported, and wrist in neutral. In the test procedure, three measurements were made for hand grip and pinch grip strength with 1-min intervals between each measurement and the averages were recorded. The value on the screen was read and recorded in pounds (1 kg = 2.2 pounds) [14, 15].

#### Wrist isometric muscle strength assessment

Wrist isometric muscle strength assessment was performed with a hand dynamometer (Lafayette Instrument Company, Lafayette, IN, USA). Patients were placed in supine, prone, and sitting positions using the positions required for the fine muscle test method defined by Lovett and without compensatory movements. Wrist flexor and extensor muscles were evaluated. The values obtained with isometric contraction were recorded in pounds. Muscle strength measurements were evaluated bilaterally, in three repetitions for 5 s. The average value of the three repetition results was recorded [16].

#### Pain threshold assessment

Pain sensitivity to pressure was assessed with a pain measuring device called an algometer. A baseline dolorimeter brand algometer device was used in the assessment. It makes pressure readings with a spring mechanism and displays the measurement result manually in Newton or Pound. Measurements were made 3 times from the “midpoint of the upper trapezius muscle” and the “C7” points between the lateral edge of the acromion and C7. The areas were marked before the measurement. The algometer was placed perpendicular to these points. Patients were asked to report the moment they first felt pain and a 30-s rest was given between measurements. The value on the screen was read, and the pain threshold was recorded in pounds [17, 18].

#### Anxiety level assessment

Anxiety level was determined with the Beck Anxiety Inventory. Beck Anxiety Inventory is a scale developed by Aaron T. Beck (1988). This scale consists of 21 questions. For each item, the patient is asked to report how he/she has felt during the past week. Items are scored as 0, 1, 2, or 3. The score range is 0–63. For the total score, < 21 is considered mild, 22–35 is considered moderate, and > 36 is considered severe. The Turkish validity study of the Beck Anxiety Scale was conducted by Ulusoy et al. [19].

### Statistical analysis

SPSS 27.0 package programmed was used for statistical analysis of the data. The variables evaluated in the statistical analysis of the study were defined with mean (X), standard deviation (SD), frequency (n), and percentage (%) values. The Pearson correlation test was used for correlation analysis. In addition, multiple linear regression analysis was performed to confirm the more detailed relationship of the significant correlation results between BECK and BMI and pinch grip strength. *p* < 0.05 was considered statistically significant.

## Results

When the demographic characteristics and physical parameters of the participants in the study were examined, it was found that the mean age of the participants was 35.49 ± 7.50 years, the mean height was 166.61 ± 7.49 cm, the mean weight was 66.71 ± 12.61 kg, and the mean BMI was 23.89 ± 3.35. When physical strength parameters were analyzed, it was found that there was a significant difference between dominant hand grip strength (77.67 ± 19.12) and non-dominant hand grip strength (72.18 ± 18.06) values, and the dominant hand was stronger. In addition, the mean Beck Anxiety Score was found to be 18.70 ± 11.83 (see Table 1).

**Table 1:** Demographic characteristics and mean values of parameters

	X ± SD
Age	35.49 ± 7.50
Height	166.61 ± 7.49
Weight	66.71 ± 12.61
BMI	23.89 ± 3.35
Occupational age	11.29 ± 7.73
BAI	18.70 ± 11.83
Dominant hand grip strength	77.67 ± 19.12
Non-dominant hand grip strength	72.18 ± 18.06
Dominant pinch strength	16.88 ± 4.47
Non-dominant pinch strength	15.81 ± 4.29
Dominant wrist flexors	57.82 ± 12.00
Non-dominant wrist flexors	58.77 ± 13.70
Dominant wrist extensors	60.75 ± 12.58
Non-dominant wrist extensors	58.26 ± 9.52
Dominant pain threshold	11.16 ± 3.24
Non-dominant pain threshold	11.39 ± 3.12

Abbreviations: *BAI* beck anxiety inventory, *X* mean, *SD* standard deviation, *BMI* body mass index

Correlation analyses revealed a positive and significant relationship between BMI and dominant and non-dominant hand grip strength (*r* = 0.485, *p* = 0.003; *r* = 0.501, *p* = 0.002). BMI also showed a strong relationship with dominant and non-dominant pinch strength (*r* = 0.511, *p* < 0.001; *r* = 0.557, *p* < 0.001). A negative correlation was found between Beck Anxiety Score and physical strength parameters, especially non-dominant hand grip strength (*r* = 0.619, *p* = 0.005) and dominant hand grip strength (*r* =  − 0.512, *p* = 0.025) and anxiety levels. Significant positive correlations were found between pain threshold and physical strength parameters, especially non-dominant wrist extensor strength (*r* = 0.283, *p* = 0.049) and dominant hand grip strength (*r* = 0.408, *p* = 0.015) were found to increase pain threshold (see Tables 2 and 3).

**Table 2:** Correlation between BMI, professional experience, and BAI and dominant upper extremity

	**BMI**		**Professional experience**		**BAI**		**Dominant hand grip strength**		**Dominant pinch strength**		**Dominant wrist flexors**		**Dominant pain threshold**	
	***r***	***p***	***r***	***p***	***r***	***p***	***r***	***p***	***r***	***p***	***r***	***p***	***r***	***p***
Professional experience	0.079	0.587	1											
BAI	− 0.105	0.602	0.090	0.654	1									
Dominant hand grip strength	0.485	0.003*	− 0.173	0.320	− 0.512	0.025*	1							
Dominant pinch strength	0.511	< 0.001*	− 0.026	0.871	− 0.321	0.109	0.787	< 0.001*	1					
Dominant wrist flexors	0.339	0.017*	− 0.030	0.839	− 0.242	0.224	0.162	0.354	0.320	0.037*	1			
Dominant wrist extensors	0.260	0.071	− 0.112	0.442	− 0.364	0.062	0.314	0.066	0.234	0.130	0.648	< 0.001*	1	
Dominant pain threshold	0.283	0.049*	0.068	0.642	− 0.135	0.502	0.408	0.015*	0.296	0.054	0.049	0.739	0.243	0.092

Abbreviations: *BAI* beck anxiety inventory, *BMI* body mass index

**Table 3:** Correlation between BMI, professional experience, and BAI and non-dominant upper extremity

	**BMI**		**Professional experience**		**BAI**		**Non-dominant hand grip strength**		**Non-dominant pinch strength**		**Non-dominant wrist flexors**		**Non-dominant pain threshold**	
	***r***	***p***	***r***	***p***	***r***	***p***	***r***	***p***	***r***	***p***	***r***	***p***	***r***	***p***
Professional experience	0.079	0.587	**1**											
BAI	− 0.105	0.602	**0.090**	**0.654**	1									
Non-dominant hand grip strength	0.501	0.002*	− **0.142**	**0.415**	− 0.619	0.005*	1							
Non-dominant pinch strength	0.557	< 0.001*	− **0.032**	**0.833**	− 0.412	0.041*	0.743	< 0.001*	1					
Non-dominant wrist flexors	0.319	0.026*	− **0.052**	**0.721**	− 0.146	0.469	0.279	0.104	0.501	< 0.001*	1			
Non-dominant wrist extensors	0.299	0.037*	**0.069**	**0.640**	− 0.430	0.025*	0.441	0.008*	0.566	< 0.001*	0.717	< 0.001*	1	
Non-dominant pain threshold	0.199	0.170	**0.119**	**0.415**	− 0.124	0.537	0.494	0.003*	0.241	0.102	0.131	0.371	0.283	0.049*

Abbreviations: *BMI* body mass index, *BAI* beck anxiety inventory

*statistical significance

**Fig. 1:**
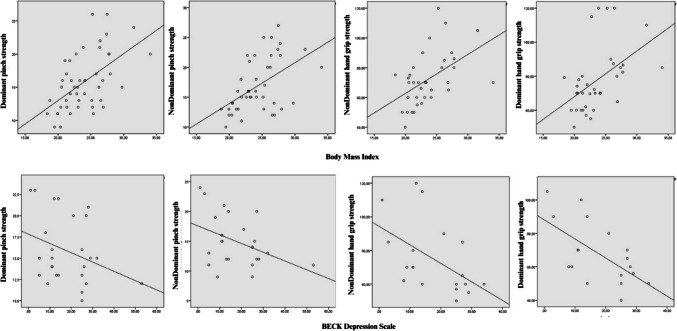
Scatter plot visualizations of multiple regression analysis

In this study, the effects of BMI and Beck independent variables on hand and pinch grip strengths were analyzed by regression analysis. In the analysis with BMI, positive and significant relationships were found on all grip strengths. In particular, the relationship between non-dominant pinch strength and BMI had the highest explanatory power (31%), and the significance level was found to be quite high (*t* = 4.494, *p* < 0.001). In addition, negative and significant relationships were found between dominant and non-dominant hand grip strengths in the analysis performed with the independent variable Beck. The explanatory rate of Beck in non-dominant hand grip strength was 38.3% and statistically significant with *t* =  − 3.249, *p* = 0.005. Although there was a negative trend between Beck and dominant pinch strength, the relationship was not significant (*p* = 0.109) (see Table 4). In addition, scatter plot visualizations of multiple regression analysis are given in Fig. 1.

**Table 4:** Regression analysis with BAI and BMI independent variables

		*R*	*R*^2^	*F*	*B*	Std.Error	Beta	*t*	*p*	VIF
BMI										
Dominant hand grip strength	(Constant)				14.326	20.066		0.714	0.480	
		0.485	0.236	10.173	2.683	0.841	0.485	3.189	0.003*	1.000
Non-dominant hand grip strength	(Constant)				10.373	18.758		0.553	0.584	
		0.501	0.251	11.083	2.618	0.786	0.501	3.329	0.002*	1.000
Dominant pinch strength	(Constant)				0.207	4.417		0.047	0.963	
		0.511	0.261	14.516	0.686	0.180	0.511	3.810	< 0.001*	1.000
Non-dominant pinch strength	(Constant)				− 1.163	3.813		− 0.305	0.762	
		0.557	0.310	20.194	0.707	0.157	0.557	4.494	< 0.001	1.000
BAI										
Dominant hand grip strength	(Constant)				94.380	8.978		10.153	0.000	
		0.512	0.262	6.036	− 1.102	0.449	− 0.512	− 2.457	0.025*	1.000
Non-dominant hand grip strength	(Constant)				88.074	7.031		12.527	0.000	
		0.619	0.383	10.556	− 1.142	0.351	− 0.619	− 3.249	0.005*	1.000
Dominant pinch strength	(Constant)				17.824	1.406		12.679	0.000	
		0.321	0.103	2.766	− 0.109	0.066	− 0.321	− 1.663	0.109	1.000
Non-dominant pinch strength	(Constant)				17.567	1.432		12.269	0.000	
		0.412	0.170	4.702	− 0.147	0.068	− 0.412	− 2.168	0.041*	1.000

## Discussion

This study aimed to investigate the relationship between professional experience and hand grip strength, pain thresholds, and anxiety levels in dentists. The results showed that there was a significant positive correlation between body mass index (BMI) and dominant and non-dominant hand grip strength. Also, anxiety level was found to have a negative effect on hand and pinch strength. We observed that physical strength parameters are closely related to the physical structure and psychological state of individuals, and anxiety levels may negatively affect physical performance. According to the literature, the effect of BMI on muscle strength is consistent with previous studies. Previous studies showing that muscle mass and body weight increase hand grip strength support our findings [20]. The negative correlation of anxiety level with muscle strength may be explained by stress-induced muscle tension and nerve-muscle interactions. It has been suggested that high anxiety levels may negatively affect muscle functions by increasing cortisol release [21].

The relationship between physical strength parameters and anxiety, pain, and physical structures in dentists is a multifaceted issue that is primarily influenced by the demanding nature of the profession. Dentists often experience work-related musculoskeletal disorders due to prolonged static postures and repetitive movements exacerbated by psychological stress and anxiety. Collectively, these factors contribute to pain and discomfort, particularly in the neck, shoulder, and lower back. The interaction of these elements emphasizes the need for a comprehensive approach to managing the physical and mental health of dental professionals [22, 23]. Dentists frequently report musculoskeletal pain, especially in the back, neck, and shoulders, with prevalence rates for musculoskeletal disorders ranging from 64 to 93% [22]. Anxiety is significantly associated with musculoskeletal pain, with higher rates of musculoskeletal disorders in the lower back when dental students have higher levels of anxiety [23]. Canova et al. reported that upper extremity functionality would decrease as hand and pinch grip strength decreased [16]. Peterson et al. found that the dominant hand had 10% more grip strength than the non-dominant hand [24]. Similarly, we found that the dominant hand had a significantly higher grip strength than the non-dominant hand in our study. Our results support the literature.

The factors that are easy to measure such as gender, age, body height, and body weight provide accurate prediction of normative grip and pinch strength [3]. Charles et al. reported a significant relationship between grip strength and BMI in a study conducted on 3522 individuals [25]. Chandrasekaran et al. also reported that hand grip strength was found to be high in individuals with high body mass index, and a significant relationship was found in these two parameters [26]. There was a positive and significant relationship between BMI and dominant and non-dominant hand grip strengths in our study. BMI also showed a strong correlation with dominant and non-dominant pinch strength. Therefore, we think that dentists with normal BMI have high hand and pinch grip strength.

The effect of upper extremity muscle strength on grip strength in dentists is important because it directly affects their functional performance and susceptibility to musculoskeletal disorders. Research shows that improved grip strength can increase the efficiency of dental procedures while reducing the risk of injury [27]. Dentists often have reduced grip strength due to prolonged and repetitive tasks, leading to pain and functional limitations. In addition, pinch force training combined with postural training has been shown to significantly improve grip strength and functional performance among dentists [27]. On the other hand, wrist extensors and flexors play an important role in providing grip force. In order for the isometric force to be released during grasping, especially the wrist extensors should be involved [14]. According to their study, Canova et al. reported that upper extremity functionality would decrease as upper extremity muscle strength and endurance decreased [16]. In our study, it was found that grip strength was high in dentists with high flexor and extensor muscle strength. We think that forearm flexor and extensor muscle strengthening programme will increase grip strength in dentists and thus improve hand functionality.

In our study, statistically significant but borderline *p* values (e.g. *r* = 0.283, *p* = 0.049) were obtained for some variables. Such results are findings that should be carefully evaluated in terms of statistical significance but may have important implications in clinical practice. For instance, the positive relationship between dominant hand grip strength and pain threshold (*r* = 0.408, *p* = 0.015) is clearly statistically significant. However, the relationship between non-dominant wrist extensor strength and pain threshold (*r* = 0.283, *p* = 0.049) has a borderline value. Although this is not very strong statistical evidence, it is noteworthy in terms of its implications for clinical practice. Clinically, this relationship suggests that strengthening the wrist extensors may be beneficial for pain management. Therefore, it may be recommended to include specific exercises for these muscle groups in rehabilitation programmes for dentists.

The repetitive movements of the upper extremity frequently used in dentists and psychological stress factors cause pain complaints in the back, neck, and shoulder region [28]. According to a study conducted in Lithuania, more than half of the dentists in Lithuania experienced fatigue and musculoskeletal complaints in various parts of their body during the 12 months of the study [29]. Zorlu et al. reported that 89.6% of dentists experienced musculoskeletal complaints at least once in their lifetime and 77.0% experienced musculoskeletal complaints in at least one body region at least once in the last 12 months [30]. The intensity, duration, and site of pain play a critical role in the physical health of individuals. Tüzün reported that physical strength parameters weakened, activities of daily living decreased, and anxiety level increased with pain that became chronic over time. Therefore, they observed a significant decrease in the quality of life [31, 32]. There were significant positive correlations between pain threshold and physical strength parameters in our study, and especially non-dominant wrist extensor strength and dominant hand grip strength stood out as factors that increased the pain threshold. Therefore, we think that the pain threshold is high in dentists with high grip and muscle strength.

The relationship between anxiety, stress levels, and muscle strength, especially grip strength, is important in dentists. Research shows that increased psychological stress is associated with increased muscle tone and prevalence of musculoskeletal disorders among dentists [22]. This interaction shows that anxiety and stress not only affect mental health but also have tangible effects on physical performance. Studies have shown that dentists experience extreme levels of stress. Myers and Myers reported that high work stress was associated with lack of exercise, poor sleep quality, alcohol and smoking, and irregular eating. In the same study, it was reported that the most common minor disorders reported by dentists in the last 14 days were irritability, nervousness or depressiveness (60%), headache (58.3%), sleep difficulties (60%), and fatigue without reason (48.2%) and that these were related to work stress [33]. Chipchase et al. investigated whether anxiety in dentists affected their decisions in the clinic and found that clinical difficulties and patients increased the level of anxiety in dentists [34]. We also found mild anxiety in the participants in our study. In addition, dentists exhibit higher muscle tone in the neck and shoulder regions, which may impair grip strength due to prolonged static postures and repetitive movements [35]. In our study, a negative correlation was found between Beck Anxiety Score and physical strength parameters, especially non-dominant and dominant hand grip strength which were negatively affected by anxiety levels. For this reason, we think that dentists with high anxiety levels have low grip strength.

The original value of our study lies in being the first to simultaneously evaluate muscle strength, grip strength, pain threshold, and anxiety levels in dentists. A review of the literature reveals no previous studies that have examined these parameters collectively in this population. In addition, our study is the first to examine the relationship between grip strength and anxiety in dentists. Therefore, it is a reference for future studies. Although a significant relationship was found between grip strength and anxiety in our study, the cause-effect relationship of this relationship is not clear. Longitudinal studies are needed to answer questions such as whether the decrease in grip strength increases anxiety levels or whether high anxiety levels negatively affect muscle strength. In particular, long-term follow-up studies should investigate whether individuals with high anxiety levels experience changes in grip strength over time or whether individuals with low grip strength have a higher risk of anxiety in the future. In addition, the effects of interventions for anxiety management (relaxation exercises, stress management programmes) on grip strength and the possible ameliorative effects of exercise programmes to increase grip strength on anxiety levels should be examined with experimental design studies. Such studies may help us to understand the mechanisms underlying the relationship between anxiety and muscle strength and to develop more effective intervention methods.

## Limitations

This study has a cross-sectional design, so the causal relationships between the variables cannot be clearly determined. Longitudinal studies may provide more information, especially in terms of examining the change in the relationships between BMI, anxiety, and hand grip strength over time. Secondly, the study was conducted only on dentists and the general stability of the results for other occupational groups or the general population is limited. Further studies in different occupational groups or age ranges may provide a broader evaluation of the findings. Thirdly, the Beck Anxiety Scale, which is measured by self-report method, was used in the study. Subjective assessments may lead to measurement errors because individuals interpret their psychological states in different ways. In future studies, it is recommended that psychological assessments be supplemented with clinical interviews or biochemical stress markers. Finally, hand grip strength is widely used as an indicator of physical performance but may not accurately reflect overall muscle strength. In future studies, the use of more comprehensive physical fitness tests including different muscle groups may contribute to a stronger interpretation of the findings.

## Conclusion

This study revealed that BMI was significantly and positively correlated with hand and pinch grip strength in dentists. This may be explained by the strengthening effect of increased muscle mass and body weight on hand strength. On the other hand, a negative correlation was found between anxiety level and hand grip strength. It is thought that muscle tension and changes in nerve-muscle interactions caused by stress may negatively affect muscle functions. This finding is consistent with previous studies showing the negative effects of anxiety on muscle strength and physical performance. It also emphasizes the importance of maintaining physical endurance and mental health in occupations where dexterity and muscle strength are critical. The effects of physical and psychological factors on occupational performance should be taken into account, especially in occupations such as dentistry, which require long-term use of fine motor skills.

## Data Availability

The data are openly available and can be provided upon request via email.
